# Spontaneous clearance of serum HCV-RNA after splenectomy in a patient with HCV-related liver cirrhosis and portal hypertension: a case report

**DOI:** 10.1186/s40792-024-01899-6

**Published:** 2024-04-22

**Authors:** Toshiro Ogata, Terufumi Sakai, Sho Shibata, Hiroki Kanno, Hiroyuki Nakane, Takeshi Aoyagi, Kazuhiro Koikawa, Yoshihiko Sadakari, Gentaro Hirokata, Masahiko Taniguchi

**Affiliations:** 1 Department of Surgery, St. Mary’s Hospital, 422 Tsubukuhonmachi, Kurume, Fukuoka 830-8543 Japan; 2 Department of Gastroenterology, St. Mary’s Hospital, 422 Tsubukuhonmachi, Fukuoka, Kurume 830-8543 Japan

**Keywords:** Liver cirrhosis, Splenectomy, Spontaneous HCV-RNA clearance, Immune function

## Abstract

**Background:**

Spontaneous clearance of chronic hepatitis C virus (HCV) is rare in adults. A T-lymphocyte response is thought to be involved in HCV-RNA clearance. Splenectomy reportedly has a beneficial effect on T cell immune function in patients with cirrhosis. To the best of our knowledge, the present report is the first to describe spontaneous clearance of serum HCV-RNA within 1 year after splenectomy in a patient with cirrhosis.

**Case presentation:**

A 55-year-old man with HCV cirrhosis was transferred to our institution with advanced pancytopenia, splenomegaly, and gastric varices. He had a 1-year history of ascites, edema, and general fatigue. The patient had a Child–Pugh score of 8 and serological type 1 HCV; the HCV-RNA level was 4.7 log IU/mL. Contrast-enhanced computed tomography showed gastric varices and marked splenomegaly (estimated spleen volume of 2175 mL). Esophagogastroduodenoscopy revealed enlarged gastric varices with no red color sign, and the varices were larger than those 1 year prior. He was diagnosed with decompensated HCV-related liver cirrhosis and portal hypertension. We considered direct-acting antiviral (DAA) therapy; however, DAA therapy was not approved in Japan for patients with decompensated cirrhosis at that time. Hand-assisted laparoscopic splenectomy was performed to improve the worsening portal hypertension. Further, we planned the initiation of DAA therapy after surgery, when such therapy would become available. DAA therapy was approved 1 year after splenectomy. At that time, we measured the HCV-RNA level before the initiation of DAA therapy; unexpectedly, however, serum HCV-RNA was not detectable, and the virus continued to disappear during the following 4 years. His liver function (total bilirubin, albumin, and prothrombin time) and pancytopenia improved during the 5 years postoperatively. The serum aspartate and alanine aminotransferase levels normalized between 1 and 5 years postoperatively. Esophagogastroduodenoscopy showed no change in the gastric varices during the 5 years after surgery. The patient remained asymptomatic and continued to do well.

**Conclusions:**

We have presented a case of spontaneous clearance of HCV-RNA after splenectomy in a patient with cirrhosis and portal hypertension. Splenectomy may be associated with disappearance of HCV-RNA based on previous reports. More cases should be accumulated and evaluated.

## Background

Hepatitis C virus (HCV) spontaneously clears in 20% to 40% of acute infections [1]. By contrast, spontaneous clearance of serum HCV in patients with chronic infection is rare, with an incidence rate of 0.11 to 0.74 per 100 person-years [2, 3]. Spontaneous clearance of HCV-RNA has also been reported after total gastrectomy [4], use of minimal immunosuppressants and prednisolone in patients with HCV reinfection after liver transplantation [5], use of immune checkpoint inhibitors in the oncology setting [6], and initiation of highly active antiretroviral therapy for human immunodeficiency virus (HIV) coinfection [7]. A T-lymphocyte response is thought to be involved in HCV-RNA clearance [3, 5, 6]. We encountered a case of HCV-related decompensated cirrhosis in which HCV-RNA disappeared spontaneously after splenectomy without antiviral treatment. Splenectomy reportedly has a beneficial effect on T cell immune function in patients with cirrhosis [8–11]. The present report describes the patient’s clinical course, changes in laboratory test results and imaging findings from approximately 1.5 years before surgery to 5 years postoperatively, and discusses the effect of splenectomy in patients with cirrhosis and HCV infection based on previous reports.

## Case presentation

**Fig. 1:**
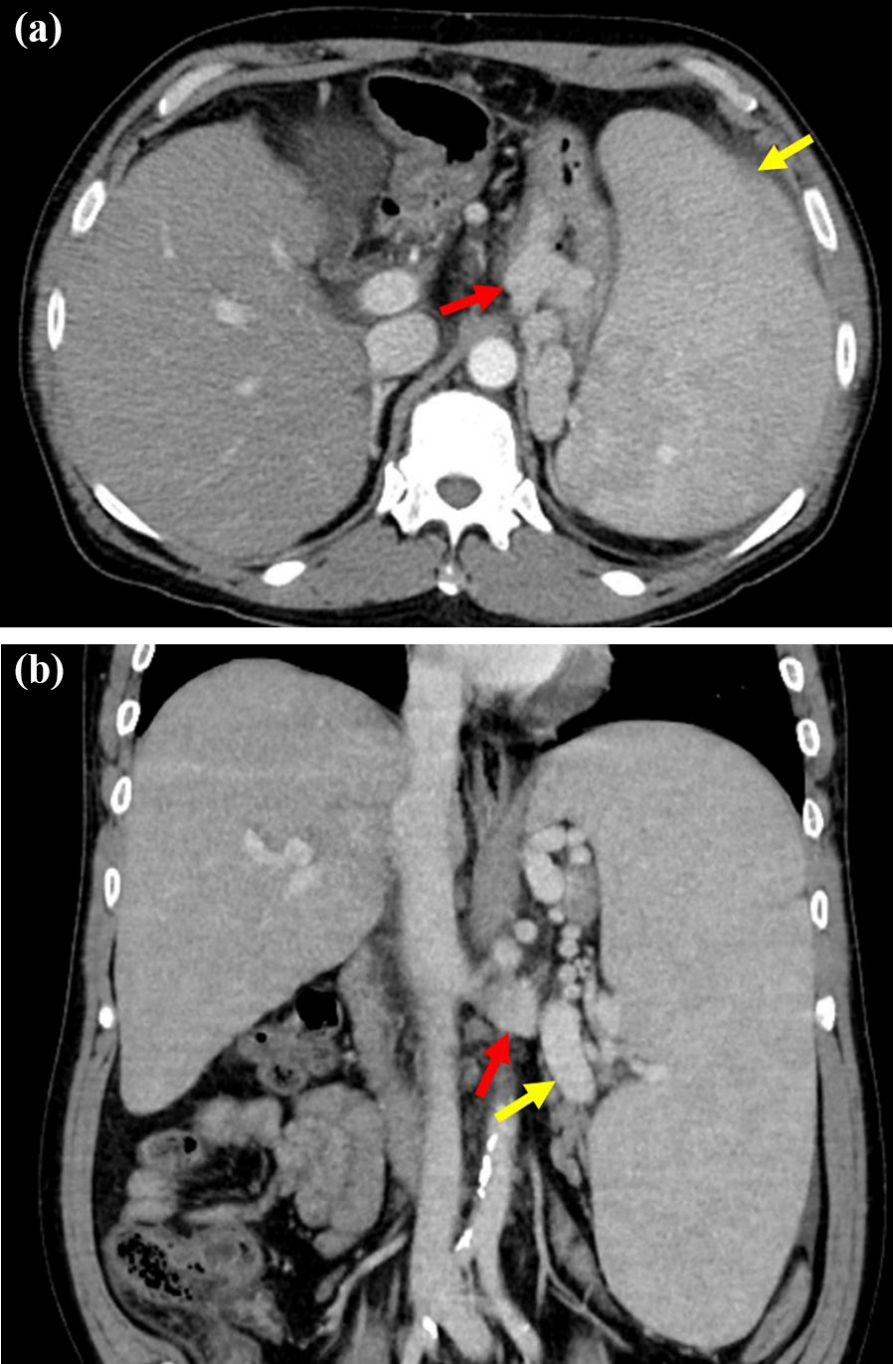
Contrast-enhanced CT before splenectomy. **a** An axial CT scan showing marked splenomegaly (yellow arrow) and gastric varices (red arrow). **b** A coronal CT scan showing an enlarged splenic vein (yellow arrow) and development of collateral vessels from the left gastric vein to a gastro-renal shunt (red arrow). *CT* computed tomography

A 55-year-old man was diagnosed with chronic HCV infection at the age of 38 years, but did not accept interferon therapy for chronic hepatitis C. He was transferred to our institution with advanced pancytopenia, splenomegaly, and gastric varices. He had a 1-year history of ascites and general fatigue. Laboratory tests revealed the following: white blood cell count, 950/μL; red blood cell count, 304 × 10^4^/μL; hemoglobin, 10.6 g/dL; platelet count, 3.7 × 10^4^/μL; albumin, 3.2 g/dL; total bilirubin, 2.5 mg/dL; and prothrombin time activity, 47.8%. Real-time polymerase chain reaction showed that the HCV-RNA level was 4.7 log IU/mL. The virus was serological type 1. Enhanced computed tomography (CT) revealed marked splenomegaly, enlargement of the splenic vein, gastric varices, and development of collateral vessels from the left gastric vein to a gastro-renal shunt (Fig. 1a, b). The spleen volume estimated by preoperative three-dimensional CT was 2175 mL. Esophagogastroduodenoscopy revealed gastric varices in the fundus of the stomach; these varices were markedly enlarged but white and showed no red color sign, and they were larger than those 1 year prior. The patient was diagnosed with decompensated HCV-related liver cirrhosis and portal hypertension. His Child–Pugh score was 8 (grade B). He was given ursodeoxycholic acid and branched-chain amino acids as liver supportive therapy. The ascites almost disappeared with the diuretic therapy. He also had type 2 diabetes mellitus and schizophrenia, which were controlled by medication. He had no history of alcohol consumption.

We initially considered direct-acting antiviral (DAA) therapy. However, DAA therapy for patients with decompensated cirrhosis was not approved in Japan at that time. Because the patient had worsening portal hypertension, we decided to perform hand-assisted laparoscopic splenectomy to improve the portal hypertension. Furthermore, we planned initiation of DAA therapy after surgery, at which time DAA therapy would be available. Informed consent was obtained from the patient after giving an explanation of the treatment, including the benefits and risks of splenectomy [12, 13]. We then administered a pneumococcal conjugate vaccination more than 2 weeks before the operation. Hand-assisted laparoscopic splenectomy with preoperative splenic artery balloon occlusion was performed because of marked splenomegaly [14]. The operation time was 358 min, and the blood loss volume was 55 mL. The patient was found to have a small abscess around the pancreatic tail on postoperative day 12, which was treated with antibiotics. The patient was discharged on postoperative day 26. He had no postoperative portal vein thrombosis. He was treated with warfarin as a prophylactic anticoagulant to prevent portal vein thrombosis until 10 months postoperatively.

**Fig. 2:**
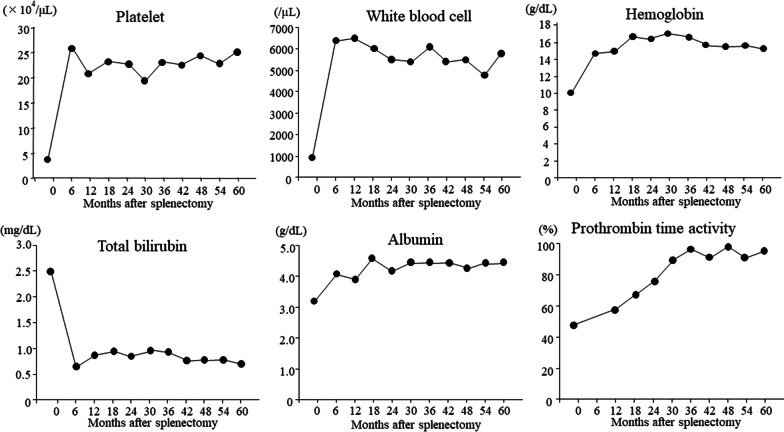
Changes in laboratory indices of liver function and blood cell counts after splenectomy. The platelet count, white blood cell count, and hemoglobin value increased between 6 months and 5 years after surgery, and pancytopenia improved after splenectomy. Total bilirubin and albumin improved between 6 months and 5 years postoperatively, and the prothrombin time increased between 12 months and 5 years after surgery

**Fig. 3:**
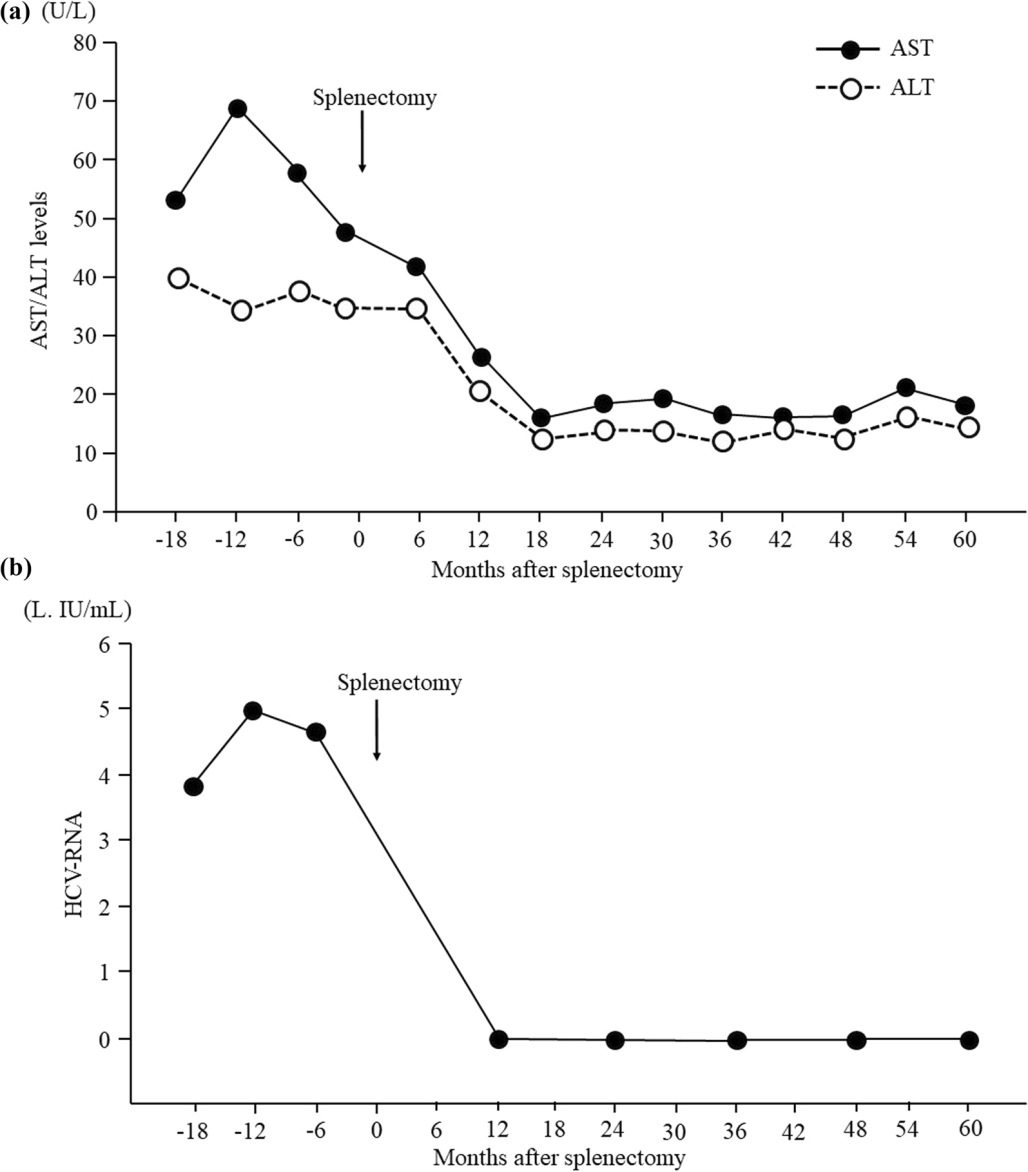
Time course of serum AST, ALT, and HCV-RNA levels. **a** The serum AST and ALT levels had decreased to within normal limits by 1 year postoperatively and remained within normal limits for a further 4 years. **b** Serum HCV-RNA was not detectable at 1 year after surgery, and disappearance of the virus continued for a further 4 years. *ALT* alanine aminotransferase, *AST* aspartate aminotransferase, *HCV* hepatitis C virus

Postoperative laboratory data showed increases in the platelet count, white blood cell count, and hemoglobin level and improvement in the pancytopenia for 5 years after splenectomy (Fig. 2). In terms of liver function, the patient’s total bilirubin, albumin, and prothrombin time also improved for 5 years after splenectomy (Fig. 2). We could not measure the prothrombin time at 6 months postoperatively because warfarin was being administered at that time. The serum aspartate aminotransferase and alanine aminotransferase levels had decreased to normal by 1 year postoperatively and were still within normal limits 4 years later (Fig. 3a). DAA therapy was approved for patients with decompensated cirrhosis at 1 year after splenectomy. At that time, we measured the HCV-RNA level before the initiation of DAA therapy; unexpectedly, however, serum HCV-RNA was not detectable. Furthermore, continuous disappearance of virus was observed during the subsequent 4 years (Fig. 3b). Esophagogastroduodenoscopy at 1, 3, and 5 years after surgery showed that the gastric varices had not changed in form and did not show the red color sign. The patient continued to do well for 5 years postoperatively.

## Discussion

Spontaneous clearance of chronic HCV is rare, with a reported incidence of 0.11 to 0.74 per 100 person-years [2, 3]. Spontaneous disappearance of HCV-RNA is associated with pregnancy [15], alcoholic hepatitis [16], hepatocellular carcinoma [3], the *IL28B* genotype [17], human leukocyte antigen-B27 [18], superinfection with hepatitis B virus [19], HIV coinfection [7], and surgical stress [4]. A T-lymphocyte response is thought to be involved in HCV-RNA clearance, but its mechanisms are not well understood [3, 5, 6, 15]. Our patient did not have hepatitis B virus, HIV infection, malignant disease such as hepatocellular carcinoma, alcohol-associated liver disease, or a history of ingesting health foods. He was not given Stronger Neo-Minophagen C (Minophagen Pharmaceutical Co., Tokyo, Japan) as liver supportive therapy because he declined the injection.

The only other report of spontaneous elimination of HCV after splenectomy was by Sekiguchi et al. [20] in 2006. They indicated that splenectomy might diminish or remit the virus burden in HCV-positive patients with cirrhosis by increasing natural killer cell activity [20]. In our patient, elimination of serum HCV occurred within 1 year after splenectomy. By contrast, Sekiguchi et al. [20] reported eradication of serum HCV-RNA between 11 and 16 years after splenectomy, which was a longer postoperative follow-up period. To the best of our knowledge, our report is the first to describe spontaneous clearance of serum HCV-RNA in the short term after splenectomy. The patient’s low viral load may have been associated with viral clearance soon after the operation in our case [2]. Splenectomy in patients with cirrhosis has produced concern over an elevated risk of infection, such as overwhelming pneumococcal sepsis [21]. However, several reports have described beneficial changes in the immune status after splenectomy in patients with cirrhosis [8–11]. According to some reports, splenectomy in patients with cirrhosis might improve their impaired immune status by increasing interferon gamma production and reducing programmed cell death protein-1 (PD-1) expression in peripheral CD4^+^ T cells or CD8^+^ T cells [8, 9]. The PD-1/programmed cell death ligand 1 (PD-L1) pathway is upregulated in chronic viral hepatitis, potentially attenuating the host’s T cell-mediated or natural killer cell-mediated antiviral immune response [6, 22]. Hashimoto et al. [8] showed that patients with HCV-related liver cirrhosis had higher levels of splenic CD4^+^ T cells as well as PD-1-expressing and PD-L1-expressing cells, and they suggested that the spleen promotes T cell dysfunction through upregulation of the PD-1/PD-L1 pathway. Splenectomy may restore impaired T cell function and natural killer cell function by blocking PD-1/PD-L1 signaling from the spleen [8, 9], which may result in increased interferon gamma production from activated T cells and natural killer cells [8–10, 22]. Our previous research also indicates that splenectomy in patients with liver cirrhosis ameliorates the impaired immune status by decreasing the numbers of suppressive cells, such as regulatory T cells and myeloid-derived suppressor cells, and increasing the population and function of effector cells, such as CD8^+^ T cells and natural killer cells [10]. Considering the findings described in the published literature [8–10], the spontaneous clearance of HCV in our patient might have been caused by a beneficial change in his immune status after splenectomy.

DAA therapy achieved a sustained virological response rate of > 95% of patients with chronic hepatitis and compensated cirrhosis in 2014 [23]. We considered DAA therapy for our patient. However, we were unable to administer this treatment because DAA therapy for patients with decompensated cirrhosis was not approved at that time (March 2018). Our patient had worsening pancytopenia, splenomegaly, and gastric varices due to severe portal hypertension. Therefore, we performed hand-assisted laparoscopic splenectomy to improve the portal hypertension. In Japan, DAA therapy was approved for patients with HCV-related decompensated cirrhosis in February 2019 [23], 1 year after our patient’s splenectomy. We observed continuous disappearance of serum HCV-RNA from 1 to 5 years after splenectomy. Therefore, the patient no longer needed DAA therapy.

Liver function and pancytopenia improved during the 5 years after splenectomy in this study. In several previous studies, splenectomy was similarly effective in improving liver function and pancytopenia in patients with cirrhosis [12, 24, 25]. Achievement of a sustained virologic response with DAA therapy has also been reported to improve liver functional reserve in patients with decompensated cirrhosis [26]. The improvement of liver function in this study was suggested to be caused by both splenectomy and elimination of HCV. We consider that the normalization of transaminase levels from 1 to 5 years after surgery was caused by elimination of HCV [27] because no reports have described long-term liver enzyme normalization by splenectomy in patients with cirrhosis.

DAA agents should be the first-choice antiviral therapy for patients with HCV-related decompensated cirrhosis and portal hypertension. However, DAA therapy is not a perfect treatment. We believe that our study is worthy of reporting because it describes a phenomenon whereby splenectomy may have a potential antiviral effect. As a limitation of this case, HCV-RNA was not measured until 12 months after splenectomy. Additionally, the patient was not examined for *IL28B* genotype or human leukocyte antigen-27 before the operation. Furthermore, the influence of surgical stress due to splenectomy cannot be ruled out.

## Conclusions

Our findings in this case suggest that splenectomy may contribute to elimination of HCV in patients with HCV-positive cirrhosis and portal hypertension. Based on previous reports, we speculate that splenectomy causes beneficial changes in patients’ immune status and is associated with disappearance of HCV-RNA. Further studies and evaluation of more patients are required to fully understand this phenomenon.

## Data Availability

The data sets generated and/or analyzed in the current study are available from the corresponding author upon reasonable request.

## Abbreviations

HCV: Hepatitis C virus
DAA: Direct-acting antiviral
HIV: Human immunodeficiency virus
CT: Computed tomography
PD-1: Programmed cell death protein-1
PD-L1: Programmed cell death ligand 1

## References

[1] Bulteel N, Partha Sarathy P, Forrest E, Stanley AJ, Innes H, Mills PR (2016). Factors associated with spontaneous clearance of chronic hepatitis C virus infection. J Hepatol.

[2] Scott JD, McMahon BJ, Bruden D, Sullivan D, Homan C, Christensen C (2006). High rate of spontaneous negativity for hepatitis C virus RNA after establishment of chronic infection in Alaska Natives. Clin Infect Dis.

[3] Minami T, Tateishi R, Shiina S, Fujiwara N, Mikami S, Sato M (2014). Spontaneous clearance of serum hepatitis C virus RNA during the clinical course of hepatocellular carcinoma in patients with chronic hepatitis C. Hepatol Res.

[4] Yoshikawa M, Morimoto Y, Shiroi A, Yoshiji H, Kuriyama S, Fukui H (2001). Spontaneous elimination of serum HCV-RNA after total gastrectomy for early gastric cancer in a patient with chronic hepatitis C. Am J Gastroenterol.

[5] Kogiso T, Hashimoto E, Ikarashi Y, Kodama K, Taniai M, Torii N (2015). Spontaneous clearance of HCV accompanying hepatitis after liver transplantation. Clin J Gastroenterol.

[6] Davar D, Wilson M, Pruckner C, Kirkwood JM (2015). PD-1 blockade in advanced melanoma in patients with hepatitis C and/or HIV. Case Rep Oncol Med.

[7] Kaung A, Sundaram V, Tran TT (2014). Spontaneous clearance of hepatitis C virus in a patient co-infected with hepatitis C virus and human immunodeficiency virus: a case report. J Gastrointestin Liver Dis.

[8] Hashimoto N, Shimoda S, Kawanaka H, Tsuneyama K, Uehara H, Akahoshi T (2011). Modulation of CD4+ T cell responses following splenectomy in hepatitis C virus-related liver cirrhosis. Clin Exp Immunol.

[9] Sumida K, Shimoda S, Iwasaka S, Hisamoto S, Kawanaka H, Akahoshi T (2013). Characteristics of splenic CD8+ T cell exhaustion in patients with hepatitis C. Clin Exp Immunol.

[10] Hirakawa Y, Ogata T, Sasada T, Yamashita T, Itoh K, Tanaka H (2019). Immunological consequences following splenectomy in patients with liver cirrhosis. Exp Ther Med.

[11] Nomura Y, Kage M, Ogata T, Kondou R, Kinoshita H, Ohshima K (2014). Influence of splenectomy in patients with liver cirrhosis and hypersplenism. Hepatol Res.

[12] Ogata T, Okuda K, Sato T, Hirakawa Y, Yasunaga M, Horiuchi H (2013). Long-term outcome of splenectomy in advanced cirrhotic patients with hepatocellular carcinoma and thrombocytopenia. Kurume Med J.

[13] Kawanaka H, Akahoshi T, Kinjo N, Harimoto N, Itoh S, Tsutsumi N (2015). Laparoscopic splenectomy with technical standardization and selection criteria for standard or hand-assisted approach in 390 patients with liver cirrhosis and portal hypertension. J Am Coll Surg.

[14] Ogata T, Mikagi K, Sakai H, Yasunaga M, Okuda K, Kinoshita H (2014). Hand-assisted laparoscopic splenectomy with preoperative splenic artery balloon occlusion for massive splenomegaly. J Jpn Soc Endosc Surg.

[15] Clohessy P, Polis S, Post J (2013). Spontaneous clearance of hepatitis C virus during pregnancy. Obstet Med.

[16] Silva MJ, Calinas F (2015). Spontaneous clearance of hepatitis C virus during alcoholic hepatitis: the alcohol killed the virus?. BMJ Case Rep.

[17] Thomas DL, Thio CL, Martin MP, Qi Y, Ge D, O'Huigin C (2009). Genetic variation in IL28B and spontaneous clearance of hepatitis C virus. Nature.

[18] Neumann-Haefelin C, McKiernan S, Ward S, Viazov S, Spangenberg HC, Killinger T (2006). Dominant influence of an HLA-B27 restricted CD8+ T cell response in mediating HCV clearance and evolution. Hepatology.

[19] Sagnelli E, Coppola N, Pisaturo M, Masiello A, Tonziello G, Sagnelli C (2009). HBV superinfection in HCV chronic carriers: a disease that is frequently severe but associated with the eradication of HCV. Hepatology.

[20] Sekiguchi T, Nagamine T, Takagi H, Mori M (2006). Reduction of virus burden-induced splenectomy in patients with liver cirrhosis related to hepatitis C virus infection. World J Gastroenterol.

[21] Okabayashi T, Hanazaki K (2008). Overwhelming postsplenectomy infection syndrome in adults - a clinically preventable disease. World J Gastroenterol.

[22] Wang XF, Lei Y, Chen M, Chen CB, Ren H, Shi TD (2013). PD-1/PDL1 and CD28/CD80 pathways modulate natural killer T cell function to inhibit hepatitis B virus replication. J Viral Hepat.

[23] Tahata Y, Sakamori R, Takehara T (2021). Treatment progress and expansion in Japan: from interferon to direct-acting antiviral. Glob Health Med.

[24] Akahoshi T, Tomikawa M, Korenaga D, Ikejiri K, Saku M, Takenaka K (2010). Laparoscopic splenectomy with peginterferon and ribavirin therapy for patients with hepatitis C virus cirrhosis and hypersplenism. Surg Endosc.

[25] Yamamoto N, Okano K, Oshima M, Akamoto S, Fujiwara M, Tani J (2015). Laparoscopic splenectomy for patients with liver cirrhosis: Improvement of liver function in patients with Child-Pugh class B. Surgery.

[26] Atsukawa M, Tsubota A, Kondo C, Toyoda H, Nakamuta M, Takaguchi K (2022). Time-course changes in liver functional reserve after successful sofosbuvir/velpatasvir treatment in patients with decompensated cirrhosis. Hepatol Res.

[27] Khan ST, McGuinty M, Corsi DJ, Cooper CL (2017). Liver enzyme normalization predicts success of Hepatitis C oral direct-acting antiviral treatment. Clin Invest Med.

